# Comment on Castillo et al. *Ganoderma zonatum* Is the Causal Agent of Basal Stem Rot in Oil Palm in Colombia. *J. Fungi* 2022, *8*, 230

**DOI:** 10.3390/jof8090941

**Published:** 2022-09-07

**Authors:** R. Russell M. Paterson

**Affiliations:** 1 Centre of Biological Engineering (CEB), Gualtar Campus, University of Minho, 4710-057 Braga, Portugal; russell.paterson@deb.uminho.pt; 2 Department of Plant Protection, Faculty of Agriculture, Universiti Putra Malaysia, Selangor 43400, Malaysia

I was very interested to read Castillo et al. (2022) as it is an area in which I have experience [1]. The authors are correct to state that identification of species is important for understanding pathogenicity. However, there are major problems.

The title of the paper only refers to ca. 20% of the work presented in the paper and is inadequate. There was no morphological information provided for the isolated specimens and so it is not known if they are even *Ganoderma*. Assuming they are, *Ganoderma miniatocinctum* was not included in the molecular analysis and yet is one of the pathogens of oil palm. *G. ryvardenii* is another potentially pathogenic species. One or more of the specimens could conceivably be these species. At least some of the isolated specimens appear to be different and the seven specimens could be interpreted as representing more than one taxon. There is inadequate information about from where the specimens were isolated, and seven specimens do not represent the diversity of *Ganoderma* causing basal stem rot in Colombia.

The taxonomy of *Ganoderma* is very complex and care needs to be taken to avoid introducing more confusion.

I would be grateful for a reply to my comments by the authors.
